# Manual blood exchange transfusion does not significantly contribute to parasite clearance in artesunate-treated individuals with imported severe *Plasmodium falciparum* malaria

**DOI:** 10.1186/1475-2875-12-115

**Published:** 2013-03-27

**Authors:** Annemarie R Kreeftmeijer-Vegter, Mariana de Mendonça Melo, Peter J de Vries, Rob Koelewijn, Jaap J van Hellemond, Perry JJ van Genderen

**Affiliations:** 1 Department of Internal Medicine, Division of Infectious Diseases, Academic Medical Center, Meibergdreef 9, Amsterdam, 1105 AZ, The Netherlands; 2 ACE Pharmaceuticals BV, Schepenveld 41, Zeewolde, 3891 ZK, the Netherlands; 3 Department of Internal Medicine, Harbour Hospital, Haringvliet 72, Rotterdam, 3011 TG, The Netherlands; 4 Institute for Tropical Diseases, Harbour Hospital, Haringvliet 72, Rotterdam, 3011 TG, The Netherlands; 5 Department of Internal Medicine, Tergooi Hospitals, Van Riebeeckweg 212, Hilversum, 1213 XZ, The Netherlands; 6 Laboratory of Parasitology, Harbour Hospital, Haringvliet 2, Rotterdam, 3011 TD, The Netherlands; 7 Department of Medical Microbiology and Infectious Diseases, Erasmus MC, Rotterdam, The Netherlands

## Abstract

**Background:**

Exchange transfusion (ET) has remained a controversial adjunct therapy for the treatment of severe malaria. In order to assess the relative contribution of ET to parasite clearance in severe malaria, all patients receiving ET as an adjunct treatment to parenteral quinine or to artesunate were compared with patients treated with parenteral treatment with quinine or artesunate but who did not receive ET. ET was executed using a standardized manual isovolumetric exchange protocol.

**Methods:**

All patients in the Rotterdam Malaria Cohort treated for severe *P. falciparum* malaria at the Institute for Tropical Diseases of the Harbour Hospital between 1999 and 2011 were included in this retrospective follow-up study. Both a two-stage approach and a log-linear mixed model approach were used to estimate parasite clearance times (PCTs) in patients with imported malaria. Severe malaria was defined according to WHO criteria.

**Results:**

A total of 87 patients with severe malaria was included; 61 received intravenous quinine, whereas 26 patients received intravenous artesunate. Thirty-nine patients received ET as an adjunct treatment to either quinine (n = 23) or artesunate (n = 16). Data from 84 of 87 patients were suitable for estimation of parasite clearance rates. PCTs were significantly shorter after administration of artesunate as compared with quinine. In both models, ET did not contribute significantly to overall parasite clearance.

**Conclusion:**

Manual exchange transfusion does not significantly contribute to parasite clearance in artesunate-treated individuals. There may be a small effect of ET on parasite clearance under quinine treatment. Institution of ET to promote parasite clearance in settings where artesunate is available is not recommended, at least not with manually executed exchange procedures.

## Background

European travellers returning from malaria-endemic areas are at increased risk of developing severe malaria. If left untreated, the disease can evolve rapidly into a life-threatening disease with a near to 100% case-fatality rate [1]. Until the year 2008, severe malaria was treated with intravenous quinine, a cinchona alkaloid which has potentially serious adverse effects. After the superior outcome of artesunate over quinine for the treatment of severe *Plasmodium falciparum* malaria became apparent in two large field trials in Asia and Africa [2,3], artesunate is also increasingly used for treating severe malaria in travellers from non-endemic countries [4], even though the discussion whether artesunate is also superior over quinine in non-immune travellers has not been settled yet [5].

In circumstances where optimal anti-malarial and supportive treatment is available, severe *P. falciparum* malaria in industrialized countries is still associated with a case-fatality rate of up to 10% [6]. In an effort to further reduce the mortality of severe malaria, exchange transfusion (ET) is often used as an adjunct therapy to reduce parasite load in cases of high parasitaemia. Besides direct removal of parasitized red cells, ET also improves blood viscosity by removal of uninfected red cells with reduced deformability, thereby reducing microcirculatory sludging and improving oxygen-carrying capacity [7]. Furthermore, ET additionally aims at removing parasitic antigens and toxic products and correction of severe anaemia. Although numerous cases in which ET was successfully used have been described, its use remains controversial mainly because randomized controlled trials have not been performed. Some researchers have cautioned against the use of ET, given the lack of conclusive scientific evidence for a survival benefit and the associated risks of fluid overload, transfusion reactions and potential transmission of blood-borne infections [8]. Much of the arguments against ET also apply to transfusion for anaemia, which is nevertheless often required during the course of severe malaria. On the other hand, many cases of survival from high parasitaemia that were successfully treated with anti-malarials alone have been documented in the literature [9-14].

In 1998, ET was added to the standard of care for patients with severe malaria at the Institute for Tropical Diseases of the Harbour Hospital in Rotterdam, with additional selection criteria over the WHO criteria for severe malaria (see below). Given the low case-fatality rate of severe malaria since then, the ET selection criteria and exchange protocol remained virtually unchanged except for replacing quinine by artesunate as the parenteral anti-malarial treatment of choice since 2008. The mainstay of treatment of severe malaria is a rapid reduction of parasite biomass. Since quinine and artesunate both induce parasite elimination but at different rates, the relative contribution of ET to parasite clearance is unknown. In the present study the contribution of ET to parasite clearance was examined by studying patients receiving intravenous quinine or artesunate only as well as patients who received ET as an adjunctive treatment on top of treatment with either intravenous quinine or artesunate.

## Methods

### Patients

All patients in the Rotterdam Malaria Cohort treated for severe *P. falciparum* malaria at the Institute for Tropical Diseases of the Harbour Hospital between 1999 and 2011 were included in this retrospective follow-up study. All patients were diagnosed with imported *P. falciparum* malaria and received intravenous anti-malarial treatment and - when indicated - ET in a well-equipped intensive care unit (ICU). In order to assess the relative contribution of ET to parasite clearance in (severe) malaria, all patients receiving ET as an adjunct treatment to parenteral quinine or to artesunate were compared with patients treated with parenteral treatment with quinine or artesunate but who did not receive ET. In 2008, artesunate became available in the Netherlands and replaced quinine as the treatment of choice for severe malaria. Therefore, four treatment groups could be discerned. Group I (Quinine only) consisted of malaria patients receiving quinine without ET. Group II (Artesunate only) consisted of malaria patients receiving artesunate mono-therapy. Group III (Quinine + ET) consisted of malaria patients receiving ET as an adjunct treatment to parenteral treatment with quinine., whereas group IV (Artesunate + ET) consisted of malaria patients who received artesunate plus ET. In order to properly evaluate the parasite clearance in relation to treatment mode, of each malaria patient, demographic, clinical, and laboratory data were collected using a standardized case report form after de-identification of individual patient data.

### Laboratory examinations

Malaria was diagnosed following standard procedures that comprise quantitative buffy coat (QBC) analysis, a rapid diagnostic test (RDT) targeting Histidine Rich Protein II (Binax NOW® Malaria Test,Binax, Inc, Maine, USA), and microscopic examination of thick and thin blood smears prepared from freshly collected blood specimens from finger punctures. Thick blood smears were stained with Field’s stain (Brunschwig Chemie, Amsterdam, the Netherlands) and thin smears were fixed with absolute methanol for 3 minutes and stained with Diff Quick (Medion Diagnostics, Düdingen, Switzerland). Parasite density was expressed as the number of *P. falciparum* trophozoites per 100 red blood cells (RBCs) in a thin film or the number of parasites per 100 white blood cells (WBCs) in a thick film. The parasite load (asexual parasites per microlitre) was calculated from these figures using the actual WBC and RBC counts. Other laboratory examinations included haematocrit (Hct), platelet (PLT) count, serum electrolytes and creatinine, liver enzymes and total bilirubin, blood glucose and plasma lactate, as described previously [15]. Additional investigations were initiated according to the clinical judgement of the attending staff.

### Definitions

#### Severe malaria

Patients were considered having severe *P. falciparum* malaria if they met predefined criteria for severe malaria as published previously [1].

#### Exchange transfusion

Patients were considered candidates for ET if they met one of the following additional criteria [16]:

•Parasitaemia of more than 10% infected RBCs

•Presence of schizonts in peripheral blood

•Indication of organ damage (serum creatinine > 250 μmol/L; systolic pressure < 80 mm Hg with cold extremities; Glasgow Coma Scale of less than 12; convulsions; pulmonary oedema; serum bilirubin > 50 μmol/L)

•Acidaemia (pH < 7.25)

•Diffuse intravascular coagulation

•Haemoglobinuria

#### Parasite clearance times

Parasite clearance time (PCT) 50%, PCT 90%, PCT 95%, PCT 99% were defined as the time (in hours) to obtain a 50%, 90%, 95%, 99% reduction in parasite burden after start of the treatment, respectively. T = 0 was defined as the time point at which the first dose of parenteral anti-malarial treatment was given.

### Treatment

#### Quinine

Quinine dihydrochloride (magistral preparation) was administered intravenously with an initial loading dose of 20 mg/kg (maximum 1,800 mg) infused over 4 hours (in 500 mL of 5% dextrose in water), followed by 10 mg/kg infused over 8 hours three times a day (maximum 1,800 mg/day) until starting oral therapy.

#### Artesunate

Artesunate (Malacef 60™, ACE Pharmaceuticals, Zeewolde, the Netherlands) was given intravenously in a dose of 2.4 mg/kg on T = 0, 12 and 24 hours later and then daily thereafter until the patient could tolerate oral medication.

#### Exchange transfusion

ET was performed using a manual isovolumetric approach as described previously [16]. The ET procedure was initiated as soon as indications of organ dysfunction or hyperparasitaemia became apparent. After admission to the ICU, a large-bore cannula was inserted in the right antecubital vein, which was used to transfuse fresh RBCs and plasma (the ‘in-flow’ tract). A second large-bore venous catheter was inserted into the antecubital vein of the opposite arm, which was used to collect blood into a donor set (the ‘out-flow’ tract). One unit of leukocyte depleted donor RBCs (approximate volume 270–290 ml, Haematocrit 0.5 – 0.65, Sanquin, Amsterdam) and 1 unit of donor fresh-frozen plasma (FFP, volume approximately 310 ml, Sanquin, Amsterdam) were given in 1 hour via the in-flow catheter; the rate of inbound blood transfusion was controlled to approximate the blood extracted from the out-flow catheter. In order to prevent volume overload particular care was given to retain an isovolumetric balance. The clinical exchange procedure aimed at initial exchange of half the estimated blood volume (~ 2.5 L) by extraction of 5 times 0.5 L whole blood from the patient and simultaneous administration of 5 units of RBCs and 4 units of fresh frozen plasma FFP after which residual parasitaemia was measured. When the parasite count had dropped below the level of 5% infected RBCs, the exchange procedure was discontinued; when the circulating parasite load remained above 10% infected RBCs, a complete procedure (5 RBCs plus 4 FFPs) was repeated; a partial exchange procedure (3 RBCs plus 2 FFPs) was executed when evaluation revealed a residual parasite load between 5 and 10% infected RBCs. Central venous pressure, blood pressure, pulse rate, oxygen saturation and urine output were monitored frequently during the ET procedure, which always took place at a well-equipped ICU.

### Estimation of parasite clearance times

Both a two-stage approach [17] and log-linear mixed effects modelling [18] were used to estimate parasite clearance times. The two-stage approach estimates individual parameters by fitting the parasite count versus time curve of each patient separately using the parasite clearance estimator provided by the WorldWide Antimalaria Resistance Network (WWARN) [17]. The population mean and variance are derived from these data [19]. This model may have a limited degree of precision when individual data are sparse. The fewer measurements per individual, the greater the estimation error which may have a profound influence on parameter estimates [20]. Furthermore, no weighting of individual data is done. In heterogeneous data sets not all individuals have the same number of measurements and some individual‘s parameters may be estimated more precisely than those of others [21]. Since this imbalance is not taken into account in the subsequent analysis, inter-individual variability tends to be overestimated [22].

To account for these imitations, the data were also analysed with a population model. With population modelling, such as with linear mixed models, all data from the entire population are analysed simultaneously, while still taking into account the correlation in the data, i.e. grouping the observations from individual patients [19]. It additionally allows for analysis of heterogeneous data (sparse and/ or imbalanced) while identifying and estimating both inter- and intra-individual variability [23]. It has the principal advantage of yielding a true “mean data curve” whereas the two-stage model approach only produces the mean of a series of individual curves. The time course of the parasite count was fitted to a mono-exponential elimination model as previously described [18]. A log-linear mixed effects model, estimating the initial parasite count and the parasite elimination rate, was generated using restricted maximum likelihood method. Based on the estimates of this model, the time to 50% (PCT_50_), 90% (PCT_90_), 95% (PCT_95_) and 99% (PCT_99_) clearance of the parasites was calculated. Treatment mode (artesunate or quinine), exchange transfusion (yes or no) and time were entered as explanatory variables, including the interaction between treatment and time and between exchange transfusion and time. To account for the repeated measurements, intercepts and slopes were allowed to change over patients. Linear mixed effects modeling was done on the logarithmic values of the parasite count using the MIXED function in SPSS.

In this study, both methods were used to estimate the role of exchange transfusion in parasite clearance since the WWARN model has recently been promoted as the method of choice for estimating parasite clearance [17]. Although both methods demonstrated the same trend, the mixed model yields a more precise estimate of inter-individual variability than the traditional two-stage approach, especially in heterogeneous observational datasets [23] as in the present study and, therefore, the results of the mixed model were preferred and shown in the main text. For reasons of clarity, the data of the two-stage approach (WWARN model) are given in Additional file 1.

### Statistics

All analyses were done with software SPSS version 18.0 (IBM Inc., Chicago, IL).

Demographic data, clinical presentations and outcome were compared between patients who were treated with quinine or artesunate alone and patients who received ET adjunctive to quinine or artesunate using Kruskal-Wallis test for non-parametric comparisons followed by Dunn. Since patients receiving exchange transfusion are more likely to present with more severe disease, statistical comparisons focused on comparisons of groups with comparable disease severity (*e.g.* comparing quinine + ET (group III) with artesunate + ET (group IV) and comparing quinine only (group I) with artesunate only (group II) on the other hand) with the use of non-parametric Mann–Whitney test. A p-value of less than 0.05 was considered significant.

### Ethical considerations

Given the retrospective observational design of this study, ethical approval of this study was not required, according to the Dutch Medical Research Involving Human Subjects Act.

## Results

### Patient characteristics

A total of 87 patients with severe malaria were included in this study; 61 received i.v. quinine, whereas 26 patients received i.v. artesunate. Thirty-nine patients received ET as an adjunct treatment to either quinine (n = 23) or artesunate (n = 16). As shown in Table 1, almost half (43 %) of the patients were non-immune European travellers. Most patients acquired their *P. falciparum* infection in West Africa and had not used chemoprophylaxis. Fifty- six patients (64%) were classified with severe malaria (one or more WHO criteria), the remaining 31 patients were given parenteral anti-malarial (24 received quinine while seven received artesunate monotherapy) because parasitaemia was between 2-5%, which is the per-protocol treatment policy at the Harbour hospital of Rotterdam. Patients receiving ET presented with more severe disease (including significantly higher parasitaemia) as compared with malaria patients not receiving ET (Table 1), in line with the clinical treatment algorithm. Among patients receiving ET, there was no difference between those treated with quinine and those treated with artesunate with regard to demographic data, ethnicity, continent of acquisition, vital signs on admission, laboratory data on admission or outcome (Table 1). Patients treated with artesunate monotherapy (group II) only differed from patients treated with quinine monotherapy (group I) in duration of hospitalization (4.4 ±1.1 days versus 6.9 ± 2.3 days respectively, p-value 0.0006).

**Table 1:** **General characteristics of patients with imported severe *P. falciparum *malaria**

	**Parenteral antimalarial treatment without exchange transfusion**		**Parenteral antimalarial treatment with exchange transfusion**	
	**(n=48)**		**(n=39)**	
	**Quinine monotherapy**	**Artesunate monotherapy**	**Quinine + exchange transfusion**	**Artesunate + exchange transfusion**
	**(Group I, n=38)**	**(Group II, n=10)**	**(Group III, n=23)**	**(Group IV, n=16)**
**Demographics**				
Age, years ^***note 1***^	39 (33–44)	42 (33–55)	48 (38–55)	52 (44–59)
Male, female, n (%) ^***note 2***^	25 (66), 13 (34)	8 (80), 2 (20)	13 (57), 10 (43)	10 (63), 6 (37)
Weight, kg ^***note 2***^	72 (62–81)	83 (75–86)	70 (60–90)	81 (72–87)
**Ethnicity,** n (%) ^***note 2***^				
Caucasian	12 (32)	5 (50)	14 (60)	7 (43)
African	20 (53)	5 (50)	5 (22)	6 (38)
Asian	4 (10)	-	2 (9)	2 (13)
Other/unknown	2 (5)	-	2 (9)	1 (6)
**Continent of acquisition**, n (%) ^***note 2***^				
Africa, [West-Africa]	34 (89), [27 (79)]	10 (100), [7 (70)]	21 (92), [17 (81)]	16 (100), [13 (81)]
Asia	3 (8)	-	1 (4)	-
South-America	1 (3)	-	1(4)	-
**Vital signs on admission**				
Impaired consciousness (%), [GCS≤11] ^***note 2***^	2 (5), [0 (0)]	1 (10), [0 (0)]	5 (22), [3 (60)]	3 (19), [1 (100)]
Body temperature, °C ^***note 2***^	38.6 (38.2-40.1)	39.1 (38.3-39.6)	38.2 (37.0-39.8)	38.9 (37.7-39.8)
Pulse rate, beats per minute ^***note 2***^	100 (90–113)	96 (90–116)	101 (91–113)	105 (100–116)
Systolic blood pressure, mm Hg ^***note 2***^	120 (113–129)	118 (111–135)	113 (95–120)	125 (100–132)
**Laboratory data on admission**				
Parasite load, parasites/μL ^***note 1***^	111,650 (35,750-185,325)	74,000 (41,400-142,350)	357,000 (162,000-514,000)	313,500 (151,950-536,675)
Schizontaemia, n (%) ^***note 3***^	11 (29)	2 (20)	14 (61)	12 (75)
Serum creatinine, μmol/L ^***note 4***^	97 (78–114)	103 (85–124)	121 (95–159)	120 (88–163)
Plasma lactate, mmol/L ^***note 5***^	1.8 (1.5-2.7)	2.3 (2.0-2.8)	2.3 (1.8-3.6)	3.0 (1.9-4.2)
**Severe malaria** (WHO 2010), n (%) ^***note 1***^	14 (37)	3 (30)	23 (100)	16 (100)
**Outcome**				
Duration hospitalisation, days ^***note 6***^	6 (1–14)^&^	5 (4–5)	9 (3–19)	9 (6–11)
Hemodialysis, n (%) ^***note 7***^	0 (0)	0 (0)	2 (9)	3 (19)
Mechanical ventilation, n (%) ^***note 7***^	0 (0)	0 (0)	2 (9)	2 (13)
Survival, n (%) ^***note 2***^	38 (100)	10 (100)	23 (100)	15 (94)^

Data are expressed as median (interquartile range) values or as indicated otherwise. GCS = Glasgow Coma Scale; & Comparison of quinine *versus* artesunate treatment: p=0.0037.

All notes given in superscript relate to the statistical analysis of all quinine and artesunate monotherapies (n=48) *versus* all regimens containing exchange transfusion (n=39). note 1 p<0.0001; note 2 p= not significant; note 3 p<0.0005; note 4 p<0.005; note 5 p<0.01; note 6 p<0.001; note 7 p<0.05. ^ One patient succumbed to a suspected pulmonary embolism 8 days after complete parasite clearance.

### Parasite clearance times

**Figure 1:**
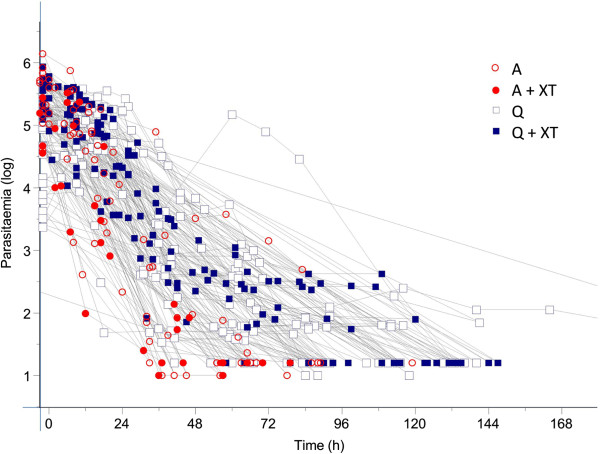
Crude data of the parasite clearance curves of patients with imported malaria treated with intravenous artesunate or quinine with or without exchange transfusion.

The crude parasite count of the 87 malaria patients and the parasite clearance in relation to time and treatment are shown in Figure 1. Data from 84 of 87 patients were suitable for estimation of parasite clearance rates. Three patients were excluded from fitting; one patient because the initial parasitaemia was too low (quinine, group I) and two others (one from the artesunate group II and one from quinine + ET group III) because the last parasite count was too high. Log-transformed data from the remaining 84 patients were fitted to a linear model and had a minimum of 4 data points. The detection limit was set at 16 (in 93% of patients) or 10 (in 7% of patients) parasites per microlitre. In six patients (three from the quinine plus ET treatment group (III), two from the artesunate group (II) and one from the artesunate plus ET group (IV)), a lag phase was identified with a median (IQR, range) duration of 9 (8.55-9.075; 4–9.5) hours. A tail, suggesting a second parasite clearance rate constant, was present in two patients from the quinine treatment group (I), while no outliers were identified.

The findings of the mixed effects model evaluating the effect of time (as a fixed effect) and treatment mode, ET and their interaction (random effects) on parasite clearance are summarized in Table 2. The Y-axis intercept (parasite load at admission) was not dependent on the treatment mode (quinine or artesunate) but, as expected, depended significantly on whether or not patients were subjected to ET. This difference is fully explained (Table 1) by the significantly higher parasite loads on admission in patients receiving ET than in patients not receiving ET (mean (95% CI) parasite count 396,568 (298,231-494,906) *vs* 122,567 (91,456-153,678 asexual parasites per μL) respectively, p value <0.0001). In contrast, in the model that estimates the parasite clearance rate, the slope of the elimination curve significantly improved when the treatment modality (quinine or artesunate) was added as a random factor but not when adding adjunctive treatment by ET or not.

**Table 2:** **Mixed effects modelling of the time course of the parasite count in severe *P. falciparum *malaria**

**Parameter**	**Estimate**	**SE**	**P-value**
**Y-axis intercept**	5.224	0.102	<0.001
**(log initial parasitaemia (LogP**_**0**_**) /μl)**			
*Adjunct treatment*	*−0.466*	*0.124*	*<0.001*^*§*^
*(no exchange transfusion)*			
*Treatment mode*	*−0.146*	*0.137*	*0.289*
*(artesunate)*			
**Parasite elimination rate constant (/h)**	−0.041	0.002	<0.001
**(parasite clearance time)**^**@**^			
*Adjunct treatment*	*0.004*	*0.003*	*0.242*^*#*^
*(no exchange transfusion)*			
*Treatment mode (artesunate)*	*−0.023*	*0.004*	<0.001*

There was no difference in initial parasitaemia between artesunate and quinine patients, but ^§^patients receiving exchange transfusion had higher initial parasitaemia than patients not receiving exchange transfusion. *artesunate had a significant effect on parasite clearance compared to quinine, ^#^while institution of exchange transfusion had no significant effect on parasite clearance. ^@^fixed effect: LogP ~ t , which reads as LogP(t) = Intercept + k.LogP_0_.

**Figure 2:**
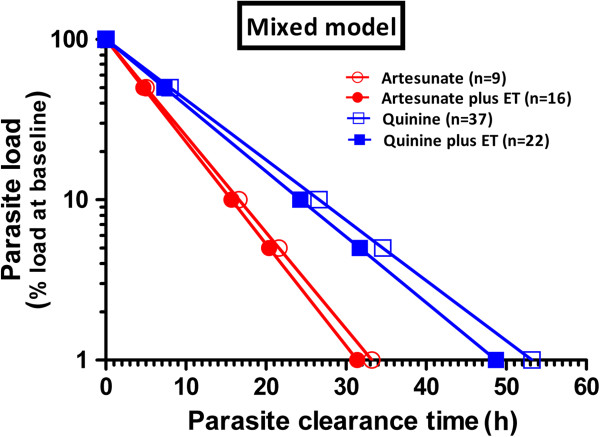
Parasite clearance curves of log transformed data according to linear mixed model analysis.

The parasite clearance times according to mixed effects model are shown in Figure 2 and Table 3. PCTs were significantly shorter after administration of artesunate as compared with quinine. In the mixed effects model the institution of ET did not improve the model, i.e. ET did not contribute significantly to parasite clearance. Interestingly, the same conclusions were also reached when data were analysed with the two-group approach model as advocated by the WWARN working group. Even though the WWARN model resulted in somewhat shorter treatment-related parasite clearance times (Additional file 1) than estimated with a mixed model, parasite clearance rate and in particular parasite elimination half-life was again significantly shorter in artesunate-treated patients than in quinine-treated patients (3.7 (95% CI 3.2-4.2) hours *versus* 6.6 (95% CI 6.1-7.2) hours, p value <0.0001, Additional files 2 and 3). Although Additional file 1 suggests that with the WWARN analysis, ET may have some beneficial effect on parasite clearance when the patient is treated with quinine, the differences in mean elimination half life between patients receiving quinine and ET (6.3 (95% CI 5.4-7.2) hours) did not differ significantly from patients treated with quinine only (6.8 (95% CI 6.1-7.6) hours, p = 0.6105). However, at some time points significant differences were observed in favour of quinine plus ET (PCT_90_ and PCT_95_) but not at PCT_50_ and PCT_99_ (Additional file 1).

**Table 3:** Parasite clearance times according to mixed model analysis of 84 malaria patients receiving parenteral anti-malarial treatment

**Treatment mode**	**Quinine (n = 59)**		**Artesunate (n = 25)**	
**Adjunct treatment**	**No exchange transfusion (n = 37)**	**Exchange transfusion (n = 22)**	**No exchange transfusion (n = 9)**	**Exchange transfusion (n = 16)**
PCT 50% (h)	8.0 (7.2-8.8)	7.3 (6.5-8.2)	5.0 (4.4-5.6)	4.7 (4.2-5.2)
PCT 90% (h)	26.6 (23.9-29.4)	24.3 (20.7-28.0)	16.6 (14.6-18.7)	15.7 (14.1-17.3)
PCT 95% (h)	34.6 (31.0-38.2)	31.7 (28.8-34.5)	21.6 (17.5-25.7)	20.4 (18.3-22.5)
PCT 99% (h)	53.2 (47.7-58.8)	48.7 (43.0-54.3)	33.2 (30.5-35.9)	31.4 (28.1-34.6)

Data are given as mean (95% confidence interval).

Three of 87 patients were excluded from analysis because of an initial parasitaemia lower than 1,000 parasites per μL (Quinine n = 1) or because the last parasitaemia exceeded 1,000 parasites per μL (Quinine plus ET n = 1; artesunate n = 1), as defined by Flegg *et al.*[17].

## Discussion

The results of this retrospective follow-up study indicate that parasite clearance is more rapid for adult travellers with severe falciparum malaria when they are treated with artesunate than with quinine. Manual exchange transfusion does not significantly contribute to parasite clearance. These conclusions were drawn irrespective of the analysis method used to estimate parasite clearance. The parasite clearance times associated with artesunate and quinine found in the present study in largely non-immune travellers are compatible with those found in field trials in endemic regions [24]. Currently, there is no evidence for artesunate resistance in returning travellers, which may be due that the majority of *P. falciparum* infections was acquired in Africa where artesunate resistance has not been described yet [25] opposed to emerging artesunate resistance in South East Asia [26].

Since its introduction in 1974 [27], ET has remained a controversial adjunct therapy for the treatment of severe malaria. Exchange transfusion has successfully been used as an adjunct therapy in malaria patients for rapid reduction of parasite burden in patients with hyperparasitaemia, improvement of the rheological characteristics of blood and removal of toxic byproducts. On the other hand, its efficacy has never been properly demonstrated in a sufficiently powered randomized controlled trial. In a meta-analysis comparing severe malaria patients who received either adjunct ET or anti-malarial chemotherapy alone, no difference in survival rates was found, although patients who received exchange transfusion were more critically ill and had significantly higher parasitaemia levels [8]. Furthermore, ET may come with significant health risks such as fluid overload, hypotension, cerebral hemorrhage, febrile and allergic reactions, metabolic disturbances and transmissible infections [28]. However, with the use of a standardized but foremost isovolumetric manual exchange protocol these adverse events were rarely observed [16].

It should be noted that the parasite clearance times may completely differ for exchange procedures using automated erythrocytapheresis. Whereas in a manual ET procedure approximately half of the blood volume is exchanged in 5 hours with an anticipated 40% reduction of parasite load in case of an isovolumetric exchange [16], an entire blood volume can be exchanged in 1.5 hours with automated erythrocytapheresis [29]. In addition, automated erythrocytapheresis may have further advantages over manual exchange procedures in terms haemodynamic stability, preservation of plasma and cellular components, efficiency and speed. However, it would only be easily available in specialized centers. In contrast, manual exchange procedures can be executed without delay once a diagnosis of severe disease has been made, even in hospitals without direct access to automated RBC exchange.

Irrespective of the exchange procedure applied, exchanging blood only removes circulating parasite stages from the circulation; a large proportion of parasitized RBCs may not be exchanged as long as they remain sequestered in the microvasculature of vital organs. In addition, exchange procedures may have additional advantages over automated RBC exchange by removing the burden of toxins and other products arising from the parasite and host response. This may explain the beneficial effect of plasma exchange in severe malaria observed in some reports [30]. Moreover, and probably underestimated, as compared to healthy subjects, parasitized RBCs but also uninfected RBCs are considerably less deformable in severe malaria [31]. Replacing less deformable RBCs by fresh RBC is a biologically plausible explanation for potential beneficial effects of exchange transfusion. Although there is currently no consensus in treatment protocol in terms of indication and volume to be exchanged, exchange transfusion was usually initiated in patients with hyperparasitaemia (either >30% or > 10% in the presence of other severe complications). Since the rapid reduction of parasite burden is mostly attributable to anti-malarial treatment, in particular artesunate, ET should not be installed for reasons of aiding in parasite clearance, at least not with manual exchange transfusion procedures.

This study is based on a single-centre experience with standardized procedure and well-equipped intensive care facilities. It has the advantage of standardized and uniform procedures but the disadvantage of a relatively small sample size. The treatment protocol reserves ET for the most severely ill patients with hyperparasitaemia and/or organ damage and this may have led to selection bias with diminishing effect modification. However, within the treatments groups subjected to ET (groups III and IV), there were no differences at baseline between artesunate and quinine treated patients and this suggest that a proper comparison is allowed. A prospective comparative trial in industrialized non-endemic countries is deemed not feasible because the number of eligible patients suffering from severe imported *P. falciparum* malaria is small, and to obtain a meaningful outcome, even in a multicentre-based study, it would require many years. It is not expected that high-level of evidence will become available soon. Since this study focused on parasite clearance alone, no conclusions can be drawn on other proposed beneficial mechanisms of exchange transfusion like removal of parasite toxins and improvement of haemorrheology.

## Conclusion

When severe *P. falciparum* malaria is treated with intravenous quinine, there may be a small beneficial effect of manual ET on parasite clearance. However, there is no benefit of ET on parasite clearance in artesunate-treated individuals. Based on these findings manual exchange transfusion is no longer recommended for patients with severe *P. falciparum* malaria who are being treated with intravenous artesunate.

## Competing interests

ARKV is PhD fellow, employed by ACE Pharmaceuticals. The other authors declare that they have no competing interests.

## Authors’ contributions

ARKV, MdMM, RK, JJvH and PJvG collected data. The data were analysed by ARKV, PJdV and PJvG. ARKV and PJvG wrote the first draft of the manuscript. The database of the Rotterdam Malaria Cohort is maintained by RK. All authors were involved in critical revision and approval of the paper.

## Supplementary Material

Additional file 1 Parasite clearance curves according to the two-stage approach according to WWARN analysis (left panel) and according to the linear mixed model analysis (right panel). Click here for file

Additional file 2 **Parasite clearance times of various modes of parenteral anti-malarial treatment.** Data are given as mean (95% confidence interval). Click here for file

Additional file 3 **Parasite clearance parameters (WWARN model) in relation to mode of parenteral anti-malarial treatment and adjunct treatment.** Data are given as mean (95% confidence interval). Click here for file
