# Supplementary figures and images for: Manual blood exchange transfusion does not significantly contribute to parasite clearance in artesunate-treated individuals with imported severe Plasmodium falciparum malaria
Source: Malar J. 2013 Mar 27;12:115. doi: 10.1186/1475-2875-12-115 (PMC3616886; doi:10.1186/1475-2875-12-115)

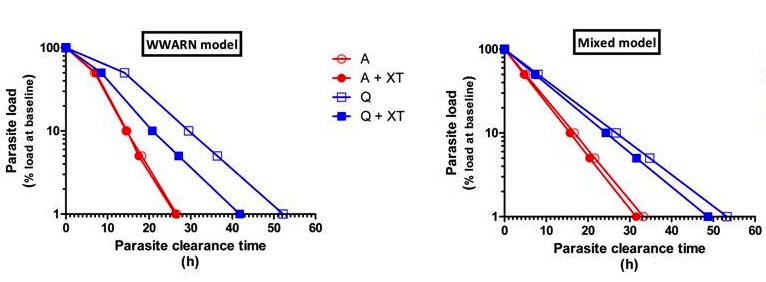

Supplement: Additional file 1 — Parasite clearance curves according to the two-stage approach according to WWARN analysis (left panel) and according to the linear mixed model analysis (right panel). [file 1475-2875-12-115-S1.jpeg]
