# Supplementary material for: Manual blood exchange transfusion does not significantly contribute to parasite clearance in artesunate-treated individuals with imported severe Plasmodium falciparum malaria
Source: Malar J. 2013 Mar 27;12:115. doi: 10.1186/1475-2875-12-115 (PMC3616886; doi:10.1186/1475-2875-12-115)
Supplement: Additional file 2 — Parasite clearance times of various modes of parenteral anti-malarial treatment. Data are given as mean (95% confidence interval). [file 1475-2875-12-115-S2.doc]

| Additional file 2. Parasite clearance times of various modes of parenteral anti-malarial treatment. Data are given as mean (95% confidence interval). | | | | |
| --- | --- | --- | --- | --- |
| Analysis  method | Parasite clearance times according to WWARN model | | | |
| Treatment regimen | Quinine  (n=59) | | Artesunate  (n=25) | |
| Adjunct treatment | No exchange transfusion  (n=37) | Exchange transfusion  (n=22) | No exchange transfusion  (n=9) | Exchange transfusion  (n=16) |
| *PCT 50* | 14.1 (8.7-19.5) | 8.5 (6.0-11.1) | 6.9 (2.6-11.2) | 7.2 (6.3-8.0) |
| *PCT 90* | 29.5 (22.9-36.1) | 20.8 (18.0-23.7) | 14.5 (10.9-18.2) | 14.7 (11.8-17.5) |
| *PCT 95* | 36.4 (29.2-43.6) | 27.1 (23.9-30.4) | 18.2 (14.5-21.9) | 17.5 (13.7-21.2) |
| *PCT 99* | 52.2 (43.5-61.0) | 41.8 (37.1-46.5) | 26.6 (22.2-30.9) | 26.3 (21.4-31.3) |
