# Supplementary material for: Manual blood exchange transfusion does not significantly contribute to parasite clearance in artesunate-treated individuals with imported severe Plasmodium falciparum malaria
Source: Malar J. 2013 Mar 27;12:115. doi: 10.1186/1475-2875-12-115 (PMC3616886; doi:10.1186/1475-2875-12-115)
Supplement: Additional file 3 — Parasite clearance parameters (WWARN model) in relation to mode of parenteral anti-malarial treatment and adjunct treatment. Data are given as mean (95% confidence interval). [file 1475-2875-12-115-S3.doc]

| Additional file 3. Parasite clearance parameters (WWARN model) in relation to mode of parenteral anti-malarial treatment and adjunct treatment. Data are given as mean (95% confidence interval). | | | |
| --- | --- | --- | --- |
|  | **Any regimen with artesunate**  **(n=25)** | **Any regimen with quinine**  **(n=59)** | P-value |
| *PCT 50 (h)* | 7.1 (5.6-8.5) | 12.2 (8.5-16.0) | P=n.s. |
| *PCT 90 (h)* | 14.6 (12.6-16.7) | 26.3 (21.9-30.6) | P<0.0001 |
| *PCT 95 (h)* | 17.7 (15.2-20.3) | 32.9 (28.2-37.7) | P<0.0001 |
| *PCT 99 (h)* | 26.4 (23.1-29.7) | 48.3 (42.5-54.1) | P<0.0001 |
| *Slope half life (h)* | 3.7 (3.2-4.2) | 6.6 (6.1-7.2) | P<0.0001 |
|  |  |  |  |
|  | **Any regimen with exchange transfusion**  **(n=38)** | **Any regimen without exchange transfusion**  **(n=46)** | P-value |
| *PCT 50 (h)* | 7.9 (6.5-9.4) | 12.8 (8.3-17.3) | P=n.s. |
| *PCT 90 (h)* | 18.3 (16.1-20.5) | 26.6 (21.0-32.2) | P=0.0063 |
| *PCT 95 (h)* | 23.1 (20.2-25.9) | 32.8 (26.6-39.0) | P=0.0076 |
| *PCT 99 (h)* | 35.3 (31.1-39.4) | 47.2 (39.6-54.9) | P=0.0159 |
| *Slope half life (h)* | 5.3 (4.5-6.0) | 6.2 (5.5-7.0) | P=0.0903 |
|  |  |  |  |
